# CarbArrayART: a new software tool for carbohydrate microarray data storage, processing, presentation, and reporting

**DOI:** 10.1093/glycob/cwac018

**Published:** 2022-03-29

**Authors:** Yukie Akune, Sena Arpinar, Lisete M Silva, Angelina S Palma, Virginia Tajadura-Ortega, Kiyoko F Aoki-Kinoshita, René Ranzinger, Yan Liu, Ten Feizi

**Affiliations:** Glycosciences Laboratory, Department of Metabolism, Digestion and Reproduction, Imperial College, Du Cane Road, London W12 0NN, United Kingdom; Complex Carbohydrate Research Center, University of Georgia, 315 Riverbend Rd, Athens, GA 30602, United States; Glycosciences Laboratory, Department of Metabolism, Digestion and Reproduction, Imperial College, Du Cane Road, London W12 0NN, United Kingdom; LAQV-REQUIMTE, Department of Chemistry, University of Aveiro, 3810-193 Aveiro, Portugal; UCIBIO, Applied Molecular Biosciences Unit, Department of Chemistry, School of Science and Technology, NOVA University Lisbon, 2819-516 Caparica, Portugal; Associate Laboratory i4HB-Institute for Health and Bioeconomy, School of Science and Technology, NOVA University Lisbon, Lisbon, 2819-516 Caparica, Portugal; Glycosciences Laboratory, Department of Metabolism, Digestion and Reproduction, Imperial College, Du Cane Road, London W12 0NN, United Kingdom; Glycan and Life Systems Integration Center (GaLSIC), Soka University, 1-236 Tangi-machi, Hachioji, Tokyo 192-8577, Japan; Complex Carbohydrate Research Center, University of Georgia, 315 Riverbend Rd, Athens, GA 30602, United States; Glycosciences Laboratory, Department of Metabolism, Digestion and Reproduction, Imperial College, Du Cane Road, London W12 0NN, United Kingdom; Glycosciences Laboratory, Department of Metabolism, Digestion and Reproduction, Imperial College, Du Cane Road, London W12 0NN, United Kingdom

**Keywords:** glycan microarray data management, glycan microarray data presentation, glycan microarray data storage, glycan microarray software, glycoinformatics

## Abstract

Glycan microarrays are essential tools in glycobiology and are being widely used for assignment of glycan ligands in diverse glycan recognition systems. We have developed a new software, called Carbohydrate microArray Analysis and Reporting Tool (CarbArrayART), to address the need for a distributable application for glycan microarray data management. The main features of CarbArrayART include: (i) Storage of quantified array data from different array layouts with scan data and array-specific metadata, such as lists of arrayed glycans, array geometry, information on glycan-binding samples, and experimental protocols. (ii) Presentation of microarray data as charts, tables, and heatmaps derived from the average fluorescence intensity values that are calculated based on the imaging scan data and array geometry, as well as filtering and sorting functions according to monosaccharide content and glycan sequences. (iii) Data export for reporting in Word, PDF, and Excel formats, together with metadata that are compliant with the guidelines of MIRAGE (Minimum Information Required for A Glycomics Experiment). CarbArrayART is designed for routine use in recording, storage, and management of any slide-based glycan microarray experiment. In conjunction with the MIRAGE guidelines, CarbArrayART addresses issues that are critical for glycobiology, namely, clarity of data for evaluation of reproducibility and validity.

## Introduction

Since their inception in 2002, microarrays of sequence-defined glycans (Fukui et al. 2002) have become essential tools in biology and medicine, particularly in elucidating glycan-binding specificities of antibodies, other proteins of the immune system, adhesins of microbial agents, and diverse other carbohydrate-recognizing systems (Rillahan and Paulson 2011; Li and Feizi 2018; Gao et al. 2019; Geissner et al. 2019; Xia and Gildersleeve 2019; Silva et al. 2021). The number and structural diversity of sequence-defined glycans that can be spotted on microarrays are increasing. Currently, they are approaching 1,000 glycans in the largest glycan microarray facilities (Glycosciences Laboratory website https://glycosciences.med.ic.ac.uk; Consortium for Functional Glycomics (CFG) website http://www.functionalglycomics.org/glycomics/publicdata/primaryscreen.jsp; National Center for Functional Glycomics (NCFG) https://ncfg.hms.harvard.edu/microarrays).

Beyond data recording, it is desirable to have dedicated software for storage, processing, and display, which includes sorting and filtering functions for the glycan probes according to structural features of those bound or not bound by particular recognition systems. Unique software tools with such functionalities were developed as prototypes (Stoll and Feizi 2009). Indeed, these have been the mainstay of data storage, presentation, and reporting in the Glycosciences Laboratory at Imperial College London (160 published and over 8,000 internal data sets). However, these software tools were developed over the years, in stages, using Microsoft Office, in house Microsoft Access databases and Visual Studio; as such, they were not readily distributable.

Encompassing the above-mentioned functionalities, we have been developing a new distributable software package using the Java programming language called Carbohydrate micro-Array Analysis and Reporting Tool (CarbArrayART). This is geared for day-to-day use by experimentalists working on slide-based glycan microarrays. An important feature of CarbArrayART is that it allows users to store centrally glycan microarray data within their laboratory with ease of retrieval, comparison, and mining of data generated. This includes compliance with the glycan microarray guidelines for Minimum Information Required for A Glycomics Experiment [(MIRAGE) (Liu et al. 2016)]. The guidelines were authored by a team of experts for generating interpretable data from a glycan microarray experiment.

**Fig. 1:**
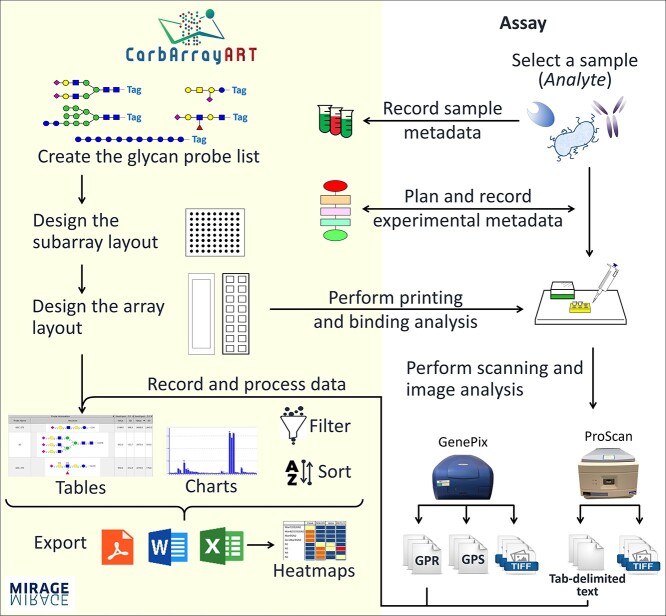
Schematic representation of the carbohydrate microarray analysis and reporting tool, CarbArrayART.

CarbArrayART benefits from the GRITS Toolbox, a software package initially developed for storing, processing, and visualizing MS data (Weatherly et al. 2019). GRITS Toolbox allows users to record metadata, such as project and collaborator information, sample metadata, and experimental protocol as well as storage related data files as archives. All these functionalities are adopted as an integral part of the CarbArrayART data storage and management system for glycan microarrays. To these, we have incorporated microarray-specific features that enable storing, processing, interpretation, and reporting of array data (Fig. 1). These include glycan probe list, array geometry information, assay conditions, and quantified array data files such as GenePix Result files, commonly referred to as GPR. Thus, CarbArrayART is the first distributable software tool that accommodates storage at a laboratory level of glycan microarray data and metadata with easy retrieval, comparison, mining, and sharing of data generated. The information generated with CarbArrayART is eminently usable by bioinformaticians and biologists using glyco-informatics tools that are discussed below.

## CarbArrayART architecture

### Workspace, project, and analyte

Workspace, project, and analyte are compartments derived from the GRITS Toolbox. When the software is run initially, users are required to create a workspace folder in which the microarray data will be saved. Project is a user-defined folder within the workspace, where microarray data files of a single or multiple analytes and the corresponding metadata are saved. For example, in the entry page for a glycan-binding sample (Analyte) users can record sample metadata compliant with the MIRAGE Glycan Array guidelines. These include the origin of the sample (synthetic, natural, or recombinant), database-associated information (e.g. PDB ID), purity, and quality control information on the sample. In the experiment design tool, users can store the experimental procedures (e.g. conditions used for different incubation and washing steps).

### Glycan probes and array geometry

Microarray laboratories have differing approaches for preparing glycan microarrays (Oyelaran and Gildersleeve 2009; Liu et al. 2012; Wang et al. 2014). CarbArrayART has been designed to cater for different slide-based array and file formats.

Glycan probe is the term used to define an arrayed glycan, its sequence, as well as the tag moiety, if applicable. Glycan sequence representations that can be used in CarbArrayART include 2D TEXT (Stoll and Feizi 2009), CFG-IUPAC, GlycoWorkbench Sequence (GWS) (Ceroni et al. 2008; Damerell et al. 2012), Web3 unique representation of carbohydrate structures (WURCS) (Matsubara et al. 2017), and the currently recommended machine-readable format GlycoCT (Herget et al. 2008). Glycans can also be imported using GlyTouCan ID (Fujita et al. 2021) and retrieving their sequence information from that repository.

Tag information is stored separately from the glycan moiety information. For example, users can designate a tag to denote its feature such as Cer32 and Cer42, which are synthetic glycolipids with ceramide having 32 and 42 carbon atoms, respectively. Inclusion the tag information facilitates comparisons of signals elicited by a given glycan sequence with different tags appended; examples are GSC-16 (NeuAcα-3Galß-4Glcß-Cer32) and GSC-18 (NeuAcα-3Galß-4Glcß-Cer42). For arrays of glycans with sequences undefined or arrays of other types of glycoconjugates, the name, source, and other informative features can be entered.

There are entry tools for: (i) generating the glycan probe list (Glycan Glyco-probe); (ii) designing the layout of spots on a block (“Subarray Layout”); and (iii) arranging positions of subarrays on a slide (“Array Layout”). There is an additional entry tool for array geometry using an Excel file, which contains the block number, spot numbers, and printing conditions for each glycan probe such as the concentrations in arrayed solution or dose arrayed per spot. This is an extended format of GenePix Array List (often referred to as a GAL file, https://www.moleculardevices.com/en/assets/app-note/br/genepix-array-list-gal-files#gref). A template and an example of this is included in the CarbArrayART software package.

### Data entry, processing, and storage

Binding signals are acquired with microarray scanners, such as ProScanArray microarray scanner (PerkinElmer) that generates a tab-delimited text file, or GenePix Microarray Scanner (Molecular Devices) that generates GenePix Result (GPR) file. In addition, GenePix Settings (GPS) are acquired, which include parameters such as the PMT voltage, scan area, identification of blocks, brightness, and contrast settings. (https://mdc.custhelp.com/app/answers/detail/a_id/18883/∼/genepix%C2%AE-file-formats). At the same time, the TIFF images of the slides are acquired.

In CarbArrayART, the scan result files such as tab-delimited file and GPR are linked to the array geometry information to generate the processed data for presentation. Other files such as GPS and TIFF image files are stored as archived data.

### Data presentation and reporting

During the image scanning, the scanner software acquires the fluorescence intensity value for each spot including mean, median, mean minus background, and median minus background. The acquired intensity values for multiple spots of each glycan probe, which are recorded in the scan result file, are averaged. Users can select the averaging method: either the mean of the replicated spots or the mean after eliminating outliers.

In the results page of CarbArrayART, the processed data, which are generated based on the scan result file and array geometry using the selected averaging method, are displayed as tables and charts.

Users can filter and sort the binding signals based on monosaccharides, or other features, e.g. sialyl linkage and oligosaccharide motifs stored in CarbArrayART. Currently, 69 types of monosaccharides and 60 types of substructures and motifs have been adapted from the Symbol Nomenclature for Glycans (Neelamegham et al. 2019) and our previous software tools.

The processed data and metadata are exportable as Excel, PDF, and Word, together with sample and experimental metadata. The exported tabulations can be used for generating heatmaps using the template file included in the CarbArrayART software package.

## Discussion and perspectives

Several web resources and software tools for glycan microarray data have been reported. Glycan Array Dashboard (GLAD) (Mehta and Cummings 2019) provides online tools to display glycan microarray results including glycans with relative fluorescence units in bar charts and heatmaps to allow users to look for glycan-binding motifs after sorting. Glycan microarray databases such as DAGR (Sterner et al. 2016), MCAW-DB (Hosoda et al. 2018), GlyMDB (Cao et al. 2020), and CarboGrove (Klamer et al. preprint posted. doi: 10.1101/2021.11.12.468378) store glycan determinants for diverse recognition systems. MotifFinder (Klamer et al. 2021) is a software tool for predicting glycan-binding motifs by introducing a new text-based glycan presentation method and algorithm for data mining. Collectively, these web resources and software tools are important for the glycan microarray community and are highly complementary with CarbArrayART.

CarbArrayART differs in being the first distributable software tool, which accommodates storage at a laboratory level of glycan microarray data and metadata with easy retrieval, comparison, mining, and sharing of data generated.

Clarity, reproducibility, and validity of glycan microarray analysis data are critical topics in glycobiology. CarbArrayART is designed to address these in conjunction with MIRAGE guidelines. The glycan-binding specificities thereby assigned pave the way to detailed studies to establish specific glycan sequences as players in molecular recognition events, for example, in many aspects of cell signaling and cell behavior, and in the initiation of infections and triggering of immunity.

In its present form, CarbArrayART is geared for slide-based glycan microarray experiments using sequence defined glycans as well as glycan fractions in glycomic-scale microarray analysis. In the future, we anticipate accommodating other microarray formats such as glycan bead array (Purohit et al. 2018), next-generation glycan microarray (Yan et al. 2019), competitive universal proxy receptor assay (Kitov et al. 2019), and liquid glycan array (Sojitra et al. 2021). Future versions of CarbArrayART, we will also include text file formats such as .csv and .xml in the export functions to enable the data to be processed further.

The first glycan microarray repository is being developed within GlyGen (York et al. 2020) with support from the NIH Glycoscience Common Fund. GlyGen is the computational and informatics resource for glycoscience to integrate data and knowledge from diverse disciplines relevant to glycobiology. Under development are new features, whereby CarbArrayART will serve as a vehicle for uploading and downloading data to and from the glycan microarray repository.

## Funding

This project is supported by Wellcome Trust Biomedical Resource grants (WT099197/Z/12/Z, 108430/Z/15/Z and 218304/Z/19/Z); March of Dimes European Prematurity Research Centre grant 22-FY18-82 and NIH Commons Fund 1U01GM125267-01.

## Conflict of interest statement

None declared.

## Availability and software for download

CarbArrayART operable in Windows 7 (64bit), Windows 10, Mac OS X Lion (10.7.5), Mac OS X Yosemite (10.10.5) and Mac OS Big Sur (11.1).

Software with online user’s manual is accessible from http://carbarrayart.org.
